# Implicancias subjetivas de mujeres adultas que recurrieron a un aborto en Argentina con posterioridad a la sanción de la Ley 27610

**DOI:** 10.18294/sc.2025.5349

**Published:** 2025-05-12

**Authors:** María Malena Lenta, Roxana Gabriela Longo

**Affiliations:** 1 Doctora en Psicología. Profesora e investigadora, Universidad de Buenos Aires. Becaria posdoctoral, Consejo Nacional de Investigaciones Científicas y Técnicas, Buenos Aires, Argentina. malenalenta@psi.uba.ar Consejo Nacional de Investigaciones Científicas y Técnicas Consejo Nacional de Investigaciones Científicas y Técnicas Buenos Aires Argentina malenalenta@psi.uba.ar; Universidad de Buenos Aires; 2 Doctora en Psicología. Docente e investigadora, Universidad de Buenos Aires. Becaria posdoctoral, Consejo Nacional de Investigaciones Científicas y Técnicas, Buenos Aires, Argentina. longoroxana@gmail.com Consejo Nacional de Investigaciones Científicas y Técnicas Consejo Nacional de Investigaciones Científicas y Técnicas Buenos Aires Argentina longoroxana@gmail.com; Universidad de Buenos Aires

**Keywords:** Salud Reproductiva, Aborto, Mujeres Cis, Feminismo, Argentina, Reproductive Health, Abortion, Cisgender Women, Feminism, Argentina

## Abstract

En Argentina, la denominada “marea verde” propició un proceso de politización del deseo y la sexualidad de mujeres y personas con capacidad de gestar, vinculadas con el reclamo del derecho a la interrupción voluntaria del embarazo (IVE), que propuso nuevas configuraciones de sentidos subjetivos. El presente trabajo tiene por objetivo analizar las implicancias subjetivas de mujeres adultas que recurrieron a una IVE en el año 2023. Desde un enfoque cualitativo e interpretativo, en intersección con la epistemología feminista y de las emociones, se realizaron siete entrevistas en profundidad a mujeres cisgénero. Se observó el impacto subjetivo del embarazo como experiencia no deseada y la persistencia de afectos como la culpa, el miedo y la vergüenza. Se identificó la posición subjetiva afirmativa ante la decisión del aborto, afincada tanto en su legalidad como en el sostén de la sociabilidad de pares vinculada a la participación en el activismo feminista y afectos como el alivio y la felicidad ante la práctica. Se hallaron obstáculos y facilitadores en el acceso al IVE en el sistema de salud para garantizar la práctica desde una perspectiva integral de cuidado.

## Abstract (en)

In Argentina, the so-called “green tide” fostered a process of politicization of women’s and childbearing people’s desire and sexuality, closely tied to the demand for the right to voluntary termination of pregnancy (VTP), which gave rise to new configurations of subjective meaning. This study aims to analyze the subjective implications for adult women who accessed a VTP in 2023. Drawing on a qualitative and interpretive approach, intersecting with feminist and affective epistemologies, seven in-depth interviews were conducted with cisgender women. The findings reveal the subjective impact of pregnancy as an unwanted experience and the persistence of affects such as guilt, fear, and shame. The women expressed a strong affirmative stance regarding their decision to have an abortion, grounded both in its legal status and in the support of peer-based sociability linked to feminist activism, as well as feelings of relief and happiness regarding the procedure. Barriers and facilitators within the healthcare system were also identified, in terms of ensuring access to VTP from a comprehensive care perspective.

## INTRODUCCIÓN

En diciembre de 2020, la aprobación en Argentina de la Ley 27610 de Acceso a la Interrupción Voluntaria del Embarazo (IVE) estableció la obligatoriedad de brindar cobertura integral y gratuita en todo el país a personas en situación de gestación que decidieran realizarse voluntariamente un aborto hasta la semana 14 y por causales (salud integral y violación) desde la semana 15. La sanción de la norma fue parte de un proceso que impulsó durante décadas el movimiento de mujeres y los feminismos^1^, que se cristalizó en el inmenso movimiento social denominado “marea verde” que se irradió en toda América Latina, promoviendo luchas por la despenalización y legalización del aborto en diferentes países de la región^2^^,^^3^. 

Este movimiento se cimentó en el trabajo de la Campaña Nacional por el Derecho al Aborto Legal, Seguro y Gratuito, una articulación surgida en el año 2005^4^ que fue precursora en la despenalización social del aborto a través de diferentes estrategias de redes en el sistema de salud^5^^,^^6^^,^^7^, el socorrismo^8^^,^^9^^,^^10^ y el sistema educativo^11^^,^^12^.

En Argentina, tal como plantean Bellucci^13^ y Rosenberg^14^^,^^15^, desde el regreso a la democracia en la década de 1980, las demandas feministas ubicaron el aborto como un derecho humano imprescindible. Pusieron el foco en la necesidad de desnaturalizar la subordinación de las mujeres y disidencias sexogenéricas para promover la apropiación y autonomía de sus cuerpos y sexualidades^16^^,^^17^; lo que para Gutiérrez^18^^,^^19^ significó una articulación entre las demandas por la libertad de decidir y la justicia reproductiva, permitiendo problematizar el modelo de maternidad obligatoria.

Como plantea Ciriza^20^, los feminismos latinoamericanos, entre los que se encuentran los argentinos, han sido críticos a las tradiciones legalistas o del feminismo liberal que hacen del derecho a decidir sobre el propio cuerpo un núcleo estratégico en el proceso de ciudadanización de las mujeres. En cambio, ponen en el aborto un foco que condensa tensiones y conflictos ligados al orden de la cultura, además del orden del derecho, y afirman que, si bien la legalización del aborto no clausura los conflictos en la subjetividad, constituye una base para el ejercicio real del derecho de ciudadanía frente a la noción de un derecho de ciudadanía abstracto. En esta línea, Ciriza propone un enfoque que empalma con los planteos de Fernández y Tajer^21^ y Rosenberg^15^ quienes señalan que el aborto y las relaciones de género están imbricadas con las determinaciones de clase, etnia y generación, lo que complejiza el abordaje del aborto y el derecho a decidir como ejercicio de la ciudadanía. Así, el bagaje de significaciones que la sociedad en general produzca acerca de las prácticas abortivas y que cada persona gestante componga en particular acerca de lo legal y lo legítimo de la decisión de abortar, juegan un papel en las configuraciones subjetivas que se organizan frente a cada situación de interrupción del embarazo, pudiendo suscitar no solo diferentes modos de significar el evento, sino también diferentes tipos afectos^21^. 

Diversas autoras^22^^,^^23^^,^^24^^,^^25^^,^^26^^,^^27^ problematizan las desigualdades en el acceso a los servicios de salud sexual reproductiva y (no) reproductiva como un elemento crítico para el proceso de ciudadanización de las mujeres y personas gestantes. El sistema sanitario tiene un rol fundamental para garantizar estos derechos y en la implementación de diversas estrategias para promover un modelo de atención y cuidado de la salud sexual integral que permita el acceso equitativo y oportuno de todas las personas a sus derechos sexuales, reproductivos y no reproductivos^28^^,^^29^. Reconocer las barreras ideológicas, religiosas, económicas y jurídicas en el acceso, las buenas prácticas y los actores claves es fundamental a la hora de diseñar y protocolizar circuitos de atención que mejoren la accesibilidad de las personas a las políticas de cuidado^22^^,^^28^. Fortalecer la accesibilidad, reducir la fragmentación del cuidado asistencial y preventivo resulta un desafío sustancial en los procesos de atención y acompañamiento en interrupciones voluntarias y legales de embarazos^23^^,^^26^. Asimismo, para una atención de la salud sexual de calidad es fundamental que se garantice la autonomía de las personas usuarias en todo el proceso y ello implica poder elegir, incluso, entre la diversidad de métodos de interrupción^25^.

Estudios como el de Jaureguiberry y Farré^30^ dan cuenta cómo el aborto es un problema de salud pública y justicia reproductiva y (no) reproductiva que demanda un rediseño institucional, y simultáneamente es un tema que se anuda a la cultura, desafiando algunos de los valores, moralidades y rituales sociales^31^. Lamas^32^ analiza la incidencia de los discursos religiosos como amenazas y prejuicios que impactan las subjetividades. Al mismo tiempo, subraya los avances tecnológicos y científicos como elementos fundamentales para que las mujeres decidan sobre sus cuerpos y vidas, mientras se transforman los significados históricos de la vida y el aborto.

Lenta *et al*.^33^ analizaron los nudos críticos identificados por psicólogas y psicólogos que participan en equipos de salud en servicios del subsistema público en el marco de la interrupción legal del embarazo. Algunos de los nudos identificados fueron los obstáculos económicos, institucionales, disciplinares y las visiones biologicistas y patriarcales que priman en los equipos de salud. Refieren a la necesidad de ampliar los dispositivos de promoción y prevención en salud sexual reproductiva y (no) reproductiva que cuestionen las modalidades de planificación en los diferentes niveles de atención, a partir de la inclusión de la perspectiva de género y derechos humanos.

Longo *et al*.^34^, desde la salud colectiva feminista, exploran tensiones, posibilidades y desafíos que enfrentan los equipos interdisciplinarios de las consejerías de salud sexual y reproductiva para garantizar el acceso a la atención en el sistema público de salud de la Ciudad Autónoma de Buenos Aires (CABA), desde la mirada de las y los trabajadores y centrándose en la implementación de la Ley 27610 de IVE. Las autoras plantean que existen cambios en las prácticas de salud a partir del nuevo escenario legislativo, y analizan el impacto de la falta de recursos en la calidad de atención, los desafíos del trabajo interdisciplinario y la integralidad de las intervenciones en salud sexual. Por otro lado, estudios como los de Santarelli y Anzorena^35^, Burton^9^^,^^10^, Atienzo *et al*.^36^ y Burton y Peralta^37^^,^^38^ han estudiado los aportes del socorrismo a las prácticas de aborto y sostienen que estos acompañamientos enfatizan en la necesidad de un abordaje feminista, de cuidado sororo y autónomo de esta práctica, lo que conlleva consecuencias sujetivantes en las personas gestantes que desean abortar, tanto en el período anterior como posterior a la sanción de la Ley 27610 de IVE en Argentina.

Numerosos trabajos vinculados a la experiencia subjetiva del aborto^14^^,^^15^^,^^21^^,^^39^^,^^40^^,^^41^^,^^42^ insisten en la necesidad de no psicologizar ni psicopatologizar la práctica del aborto, en tanto su práctica no implica necesariamente una experiencia traumática en sí, sino que ello depende del lugar que el aborto ocupa en la experiencia subjetiva de las mujeres. Tajer^43^ y Rosenberg^14^ resaltan que lo que muchas veces puede volverse traumático es el embarazo involuntario, inesperado o no deseado, al mismo tiempo en que su interrupción podría significar el cese de un padecimiento o malestar subjetivo e, incluso, una oportunidad para la reafirmación de la autonomía, tal como lo sostienen Pistani y Ceccato^44^.

Fernández y Tajer^21^, Petracci *et al*.^45^, Vaggione *et al*.^46^, Hernández-Rosete y Hipólito^47^ y Pistani y Ceccato^44^ convergen en el planteo de que la clandestinidad de la práctica del aborto es lo que suele constituirse en un elemento traumatizante o productor de sufrimiento psíquico, culpa y vergüenza, por la condena moral, social y familiar, además de los riesgos jurídicos de la ilegalidad. En este sentido, Danet Danet^48^ resalta las dificultades que atraviesan las mujeres que realizaron un aborto, el proceso de toma de decisiones y la relación con el entorno y la experiencia del estigma.

Williams Filgueiras^49^, desde una mirada psicoanalítica, analiza el proceso subjetivo vivenciado por mujeres que practicaron voluntariamente un aborto participando en la Consejería Integral en Salud Sexual de un centro de atención primaria de CABA y concluye que el aborto voluntario no es un acto neutro, tiene consecuencias y efectos singulares para cada mujer. Considera que existen algunos elementos del proceso que producen o potencian el sufrimiento psíquico y otros, en cambio, que generan alivio subjetivo. 

Asimismo, solo el trabajo de Espinosa Casanova^50^ investigó sobre los procesos de apropiación subjetiva del derecho a la interrupción voluntaria del embarazo (IVE) y la interrupción legal del embarazo (ILE) de mujeres que transitaron la práctica en el sistema de salud pública en la ciudad de Rawson, Chubut, e identificó que las mujeres que realizaron una IVE tenían un conocimiento formal del derecho al aborto, y el ejercicio de este derecho era acompañado del reconocimiento de sí como sujetas de la decisión de continuar o no con el embarazo. Aparecen en las mujeres dos hechos centrales que hacen sentido en las propias vivencias respecto al ejercicio de las IVE: las militancias y la conquista nacional de la ley de aborto. 

Si comprendemos a las experiencias en el sentido de González Rey^51^ como a un proceso continuo de iteración entre el sujeto y su entorno social que ocurre por la mediación de herramientas culturales como el lenguaje, las prácticas sociales, las tradiciones y las instituciones, podemos advertir su incidencia en la producción de subjetividad y sentidos subjetivos. Desde este enfoque, la subjetividad es comprendida como un sistema de producción simbólica-emocional, siempre contextual y situado sociohistóricamente. Mientras que los sentidos subjetivos son las configuraciones de sentido que cada sujeto construye a partir de sus experiencias concretas en contextos sociales específicos, de modo tal que no solo permiten interpretar el mundo, sino que también le otorgan un sentido emocional, simbólico y práctico que inciden en las formas de actuar y sentir^51^. De este modo, los sentidos subjetivos están ligados a las trayectorias de vida singulares y a los procesos de participación social como ha sido el caso de la “marea verde”, pero no de una manera determinista o esencialista. Las producciones de sentidos pueden configurar diversos tipos de afectos y significación ante experiencias similares en este tiempo histórico.

Ante las transformaciones normativas, institucionales y sociales a partir de la legalización del aborto en Argentina surgen interrogantes acerca de ¿cuáles son las implicancias subjetivas de las experiencias de aborto de mujeres adultas que se realizaron dicha práctica posteriormente a su legalización? ¿Cómo toman la decisión de abortar? ¿Qué emociones persisten y cuáles emergen en este escenario? ¿Cuáles son los obstáculos y facilitadores para acceder al aborto?

En este contexto, la presente investigación tiene como propósito aportar conocimiento sobre el acceso al derecho efectivo a la IVE y a la ILE y su objetivo es conocer las experiencias de aborto y sus implicancias subjetivas en mujeres adultas cisgénero de mediana edad que se realizaron abortos voluntarios posteriormente a la sanción de la Ley 27610 en Argentina y que tuvieron vinculaciones con el movimiento de la “marea verde”, al estar en contacto con la Campaña Nacional por el Derecho al Aborto Legal Seguro y Gratuito.

## MATERIAL Y MÉTODOS

En función del objetivo planteado, se desarrolló un estudio interpretativo^69^ cualitativo^52^ en intersección con la epistemología feminista^53^ y la epistemología de las emociones^54^. Para González Rey^51^, en la investigación cualitativa, las configuraciones subjetivas singulares integran lo histórico y lo diverso del contexto en una producción de sentidos y significados situados temporal y espacialmente. Esa compleja trama de realidades creadas se objetiva, ganando autonomía de los procesos que las engendraron, y logrando cierta condición de externalidad en relación con quienes viven en esa realidad, de modo que pueden ser estudiadas como producciones culturales.

Solidariamente a este enfoque de investigación cualitativa, que problematiza tanto las perspectivas empiristas como las idealistas, desde la epistemología feministas se acentúa la crítica al sesgo androcéntrico de la ciencia, a la par del desarrollo de la noción de experiencia y el valor del conocimiento situado^53^^,^^55^. De este modo, esta investigación enfatiza la relevancia del problema estudiado en el contexto de los aportes de los feminismos latinoamericanos y pone el foco en las experiencias de las participantes no solo desde lo particular de la experiencia individual, sino desde el reconocimiento de las huellas histórico-sociales en la producción de emociones, cogniciones y prácticas^56^. En este sentido, la epistemología de las emociones supone que éstas (la empatía, el compromiso, etc.) contribuyen a la comprensión y al conocimiento desde una praxis dialógica^54^ y a una aproximación constructivo-interpretativa^52^.

En este marco, se realizó el estudio con mujeres cisgénero de mediana edad del Área Metropolitana de Buenos Aires, Argentina, que se realizaron abortos entre diciembre de 2022 y agosto de 2023. A partir de una primera referencia aportada por la Campaña Nacional por el Derecho al Aborto Legal Seguro y Gratuito, las restantes mujeres fueron seleccionadas mediante un muestreo de bola de nieve^57^ y el tamaño muestral quedó delimitado por la saturación teórica. Por la complejidad de la problemática abordada en el estudio y en función de que la participación permitiera producir datos intensivos acerca del problema investigado, en primer lugar, se realizó un primer contacto telefónico con cada persona y se les explicó el tipo de participación requerida. En un segundo momento, con quienes decidieron participar, se acordó la realización de una entrevista presencial o virtual de aproximadamente 1 hora y 30 minutos de duración, según la preferencia de cada participante. Finalmente, se implementaron entrevistas en profundidad^57^, las cuales fueron grabadas y cuyos tópicos centrales correspondieron a: características sociodemográficas de las participantes, contexto del embarazo, proceso de toma de decisión con relación al aborto, tipo y modalidad del aborto, accesibilidad al aborto, afectaciones vinculadas al embarazo y al aborto, y redes de apoyo.

La muestra quedó conformada por siete mujeres heterosexuales con un rango de edad de entre 24 y 38 años y situación económica de medianos ingresos. Todas trabajaban (tres en situación precarizada y cuatro no precarizada), tres ya tenían al menos un hijo o una hija y dos ya habían tenido otras experiencias de aborto. Cuatro tenían pareja estable y tres no, una vivía sola, dos convivían con sus familias de origen, tres convivían con sus hijos e hijas y una convivía con su pareja. Asimismo, cinco accedieron al aborto en el sistema público de salud, una mediante obra social y una a través del socorrismo. Las interrupciones del embarazo se llevaron adelante mediante dos métodos: seis con método medicamentoso (con misoprostol y sin otras opciones) y una con aspiración manual endouterina (AMEU).

En todos los casos se suscribió un consentimiento informado y esclarecido de manera escrita en el que se informaron los derechos a las participantes, así como los resguardos en cuanto a la confidencialidad de los datos y la anonimización de la información. Dicho consentimiento fue aprobado por el Comité de Conductas Responsables en Investigación de la Facultad de Psicología de la Universidad de Buenos Aires, bajo el registro número CEI212022 y financiado por Universidad de Buenos Aires Ciencia y Técnica. Este artículo es parte del proyecto “Territorios de precarización: praxis instituyente de lo común y procesos de transformación psicosocial”, financiado por la Universidad de Buenos Aires, a través del Programa UBACyT.

Para el análisis de los datos se apeló a la estrategia de análisis de contenido temático^58^. Se trata de un método propicio para el abordaje cualitativo de los datos ya que se utiliza para explorar y comprender el significado de un conjunto de datos, generalmente texto, de manera sistemática y rigurosa. En este estudio, el proceso implicó diferentes fases iterativas. La primera fase fue de familiarización con los datos para identificar patrones, temas o tendencias iniciales desde una visión general que permitió identificar tres diferentes momentos vinculados a la experiencia de aborto: la noticia acerca del embarazo, la decisión del aborto y la práctica del aborto en sí, que tuvieron características centralmente descriptivas en las narrativas de las participantes, mientras que un cuarto aspecto fue la resignificación de los diferentes momentos de la experiencia de aborto a partir de las narrativas reflexivas producidas en la entrevista.

La segunda fase implicó otra lectura de los datos para la identificación de unidades de sentido organizadas a partir de códigos vinculados a temas. Para el tema “noticia acerca del embarazo” se emplearon los códigos “tipo de embarazo”, “connotación del embarazo” y “confirmación del embarazo”. Para el tema “decisión del aborto” se trabajó con los códigos “motivo de la decisión del aborto”, “modo de toma decisión del aborto” y “otros en la toma de decisión del aborto”. En cuanto al tema “práctica del aborto” se consideraron los códigos “facilitadores del aborto” y “obstáculos del aborto”. Asimismo, la recuperación de los aspectos más reflexivos acerca de la experiencia de aborto permitió configurar dos temas vinculados entre sí, y transversales a los diferentes momentos: “sentidos” y “afectos”. En cuanto a los sentidos, se codificaron las “significaciones acerca de la experiencia de aborto”. Respecto de los afectos, se implementaron códigos como “afectos negativos sobre la experiencia de aborto” y “afectos positivos sobre la experiencia de aborto”.

Por último, a partir de una tercera lectura, se elaboró el informe de resultados que permitió organizar la relación entre los códigos y los temas a partir de la elaboración de interpretaciones basadas en la descripción de los datos, las que se organizaron en los siguientes ejes: “enterarse del aborto: lo inesperado”, “llegar al aborto: tomar la decisión”, “acceder al aborto: obstáculos y facilitadores” y “sentidos y afectos sobre la experiencia de aborto: entre cambios y persistencias”.

## ANÁLISIS Y RESULTADOS

### Enterarse del embarazo: lo inesperado

En todos los casos, al indagar sobre el embarazo, las mujeres indicaron que se trató de un embarazo no intencional en el marco de vínculos de pareja o vínculos sexoafectivos estables. El embarazo fue connotado en las diferentes narrativas como un acontecimiento “no buscado”, “*no esperado*” o “*sorpresivo*” para el momento vital de cada una de ellas.

Las narrativas convergen en que el registro del embarazo fue configurándose paulatinamente a partir de cambios en el cuerpo y en las sensibilidades durante las primeras semanas por “*cambios en el cuerpo*” y la “*percepción de transformaciones y sensaciones corporales nuevas*”.

No obstante, la constatación del embarazo apareció a partir de una prueba o test:

“*Estaba muy sensible y con dolor. Me daban asco distintas comidas*. […] *Ya había averiguado en salitas* [centros de salud barriales]. *Y me dije:* ‘*¿no, me estoy adelantando?, vamos paso a paso*’*. Yo tengo una amiga que había tenido una interrupción de embarazo. Entonces, le pregunté a ella, porque ella lo hizo bastante rápido. El test me dio positivo al toque*”. (Entrevista H, 24 años)

“*A mí no me vino, yo soy muy regular. Cuando no me vino, el cuerpo viste una lo va notando, claro. Entonces me hice un test solo de embarazo y enseguida dio positivo*”. (Entrevista J, 35 años)

### Llegar al aborto: tomar la decisión

Al momento de la toma de decisión sobre el embarazo “*sorpresivo*”, los motivos de la interrupción fueron centralmente de dos tipos. Por un lado, en las narrativas de quienes no tenían hijos o hijas argumentaron que su motivo era “*no estar preparada para la maternidad*” o “*no es el momento*”. Por otro lado, quienes sí los tenían, apelaron a motivos como la situación económica inoportuna o las cargas actuales del cuidado: “*no tengo recursos económicos*” o “*priorizar a los hijos que sí tengo*”.

Sin embargo, todos los casos coincidieron en que la decisión sobre la interrupción del embarazo no les había resultado difícil ya que tenían una posición previa favorable al derecho al aborto y, en ocasiones anteriores, habían podido imaginarlo como una opción posible:

“...*yo no tenía problemas con el aborto*”. (Entrevista S, 38 años)

“...*es mi decisión sobre mi propio cuerpo, yo decidí*” (Entrevista E, 36 años)

“*...significó pensar en mi autonomía*”. (Entrevista H, 24 años)

“*...estaba a favor del aborto antes y no tuve problemas en decidir la interrupción*”. (Entrevista J, 35 años)

Esta posición afirmativa en relación con la decisión de abortar también connotó que, aunque todas sostenían haber tomado la decisión solas, no significó una sensación de soledad o la falta de apoyo, sino una posición subjetiva de autonomía en relación con el cuerpo, el propio deseo y proyecto de vida. Pues en el proceso de decisión estuvieron presentes otras personas:

“*No, lo decidí yo. Se lo conté y él me apoyó, pero lo decidí yo sola. Es mi cuerpo*”. (Entrevista A, 26 años)

“*Yo lo decidí. Mis amigas estuvieron ahí porque son un lugar seguro*”. (Entrevista F, 26 años)

“*No lo quise consultar con nadie porque sabía que era algo que tenía que resolver yo, conmigo* [...] *Mis amigas me acompañaron y también mi pareja*”. (Entrevista E, 36 años)

Si bien fueron las mujeres las que tomaron la decisión de manera autónoma, las “*amigas*” y, en algunos casos, “*las parejas*” ocuparon un lugar de acompañamiento y apuntalamiento de la decisión y del proceso de abortar. De las amigas, las mujeres esperaron espacios seguros para reflexionar y pensar sobre la toma de decisión sin juzgamientos, así como también, transmisión de otras experiencias de aborto:

“*...le pregunté a mi amiga que ya lo había hecho cómo era. Me ayudó a pensar*” (Entrevista H, 24 años)

“*...me generan seguridad tenerlas*” (Entrevista F, 26 años)

De sus parejas, las mujeres requirieron apoyo ante la decisión tomada, enfatizando un lugar de menor relevancia que ellos también asumieron, según las narrativas. 

Cabe señalar que, en los relatos, las mujeres indicaron no querer compartir el momento de la toma de decisión con familiares, especialmente con madres y padres, incluso en los casos en los que compartían la vivienda. Las mujeres que tenían trabajo formal evitaron pedir licencia para realizarse la práctica y eligieron el fin de semana para llevar adelante la interrupción del embarazo.

### Acceder al aborto: obstáculos y facilitadores

Al momento de acceder a la práctica del aborto, se pudieron identificar diferentes obstáculos y facilitadores para su concreción. En primer lugar, se destaca como facilitador el hecho de que el aborto fuera legal en Argentina al momento de solicitar la interrupción:

“*Aunque no sabía muy bien cómo era lo que había que hacer, que sea legal para mí era un montón. Me dejaba más tranquila*”. (Entrevista E, 36 años)

“*Yo sabía lo que decía la ley porque, de alguna manera, siempre estuve vinculada al movimiento del aborto, la marea verde y todo. Sabía que te lo podías hacer en un centro de salud, en la obra social, gratis. Eso fue bueno para la parte de llegar a hacerlo*”. (Entrevista S, 38 años)

La información con la que contaban las mujeres acerca de la legalidad del aborto, así como aspectos generales sobre los métodos, lugares de acceso y complejidad de la práctica fueron favorecedores de la accesibilidad. Sin embargo, en el proceso se encontraron con diferentes obstáculos propios del sistema de salud y/o de los equipos que debían garantizar las interrupciones:

“…*falta de presupuesto respecto a esa área en específico*, […] *una de las máquinas para hacer ecografías se rompió. Entonces me lo tenían que hacer de otra manera, como que también es medio complicado por las cosas que faltan. Las dificultades en el sistema de salud en todas sus ramas*”. (Entrevista A, 26 años)

“*En la salita me aclararon que cuando entregás la orden para hacerte la ecografía no te tienen que mostrarte el ultrasonido, ni dejar que escuches nada. Cuestión que eso no pasó en el lugar donde yo me fui a hacerme la ecografía* […] *Tenía varias posibilidades en ese lugar de la misma salita que demoraba más, hacer la cola en un hospital general que en realidad demoraba muchísimo más. También podía hacer por la obra social* [Obra Social de los Empleados de Comercio y Actividades Civiles (OSECAC)] *que tarda un montón de tiempo en darte un turno*”. (Entrevista J, 35 años)

“*Ponen muchas trabas y no solo eso asustan mucho a las chicas. Yo tengo 30 años, tres nenes y me decían cosas como si yo nunca hubiera tenido un hijo y cosas feas que a cualquiera le daría miedo, entonces como que atrasa* […] *Yo en mi cabeza estaba loca, porque era algo que no quería. En un momento pensé que no me lo iban a solucionar, porque me daban tantas vueltas:* ‘*que vení mañana, que vení pasado, que tenés esto y esto otro*’”. (Entrevista M, 30 años)

La falta de turnos, así como la insuficiencia de insumos, equipos e infraestructura adecuada propia de la crisis del sistema de salud, se combinaron, en varios de los casos, con información inadecuada por parte del propio sistema y, en otros, con el maltrato y la violencia deliberada ejercida por diferentes instancias, desde la administración hasta los equipos de salud, lo que implicó un superlativo malestar connotado por parte de las mujeres como “*injusto*”, “*disciplinador*” y/o “*amedrentador*”. Sin embargo, también se identificaron narrativas en las que el equipo de salud actuó de manera contenedora y cuidadosa, facilitando la práctica:

“*La consejería me dio mucha tranquilidad. Me explicaron todo. Todas las veces que pregunté. Me aclararon todo y las médicas fueron muy amorosas*”. (Entrevista E, 36 años)

“*Teníamos un trato preferente en el CESAC* [centro de salud y acción comunitaria]. *La administrativa nos permitía pasar más rápido a las que éramos de salud sexual. Se notaba el compromiso con el tema del aborto, del derecho al aborto. Había carteles y todo en las carteleras*”. (Entrevista A, 26 años)

“…*las trabajadoras, porque eran todas mujeres, fueron muy detallistas, tuvieron mucha paciencia conmigo, me trataron re bien* […] *tenían un trato* […] como muy personal con cada paciente”. (Entrevista J, 35 años)

Especialmente, los equipos y consejerías de salud sexual con perspectiva feminista y acompañamiento sororo favorecieron la accesibilidad a la práctica, aun con la falta de insumos. Y, en particular, quien recurrió a la práctica mediante el Socorrismo, indicó un cuidado y acompañamiento amoroso en relación con el proceso de la interrupción.

Asimismo, en algunos casos, también se registraron “retrasos” en relación con los tiempos esperados por las mujeres para realizarse el aborto:

“*…es un proceso en el cual el cuerpo también se va moldeando, esto fue en abril. Y finalmente el 24 de mayo me dieron el alta* […] *Estuve casi dos meses con todo*”. (Entrevista J, 35 años)

“..*.sabía que había que esperar porque no era la semana para que saliera bien con el misoprostol. No era complicado que se atrasara la ecografía. Pero igual quería ya resolverlo todo*”. (Entrevista A, 26 años)

Como se observó en las narrativas, la urgencia por interrumpir el embarazo no siempre coincidió con los tiempos del sistema de salud e, incluso, del embarazo mismo. Ello llevó a la reflexión acerca de la urgencia subjetiva de ponerle fin al embarazo.

### Sentidos y afectos sobre la experiencia de aborto: entre cambios y persistencias

Al indagar sobre la producción de sentidos y afectos vinculados a estas experiencias de aborto, resalta la persistencia de cierta *clandestinidad* o *secreto* sobre la práctica. Pues en las narrativas satura que la experiencia fue compartida con pocas personas: desde el estar embarazada, pasando por la decisión de interrumpir el embarazo hasta la propia experiencia de aborto:

“*Yo no lo hablé con nadie. Solo con mi pareja, comparaba las fechas con él* […] *Sí, imagínate que no se enteró nadie, solamente él, mi amiga y mi otra amiga. Fue todo muy rápido y yo actúe rápido también*”. (Entrevista H, 24 años)

“*Yo no quería que se enterara mi familia, mis padres. No sé lo que iban a decir. Pero preferí que no, hacerlo sin que sepan, aunque vivo en la misma casa. Solo mis amigas y mi pareja lo sabían. Me costó hablarlo seriamente. Solo podía hacer chistes con ellos*” (Entrevista F, 26 años)

“*Yo no me sentí juzgada por nadie, digamos de las personas a las que yo se lo conté. Porque elegí solo hablarlo con pocas personas*”. (Entrevista A, 26 años)

Asimismo, se identificaron afectos como el miedo, la culpa y la vergüenza. El miedo fue vinculado al desconocimiento inicial sobre la práctica, al dolor que esta podría generar e, incluso, a la muerte:

“…*me daba miedo, miedo hacerlo yo misma*”. (Entrevista A, 26 años)

“*No, no, no. Estaba totalmente decidida. Miedo no, miedo al dolor capaz, a no saber bien cómo era. Además del dolor, no saber cómo iba a ser, porque nunca lo había experimentado*”. (Entrevista H, 24 años)

“*Sí, tenía miedo de que no resultara. Tenía miedo de morirme desangrada, tenía miedo de tener que estar en mi casa. Tenía miedos impuestos por ellos*”. (Entrevista M, 30 años)

La culpa, en cambio, se vinculó con el haber quedado embarazada

“*...teniendo todos los recursos para evitarlo*”. (Entrevista S, 38 años)

“*...sin cuidarme*”. (Entrevista M, 30 años)

“*...sabiendo que podía pasar*”. (Entrevista J, 35 años)

“...*por tonta*”. (Entrevista F, 26 años).

Ello implicó la vergüenza como emoción concomitante a la posibilidad de ser juzgadas por la familia y/o la sociedad por el embarazo no intencional o inesperado. No obstante, en la mayoría de los casos, el embarazo inesperado, devenido en aborto voluntario, implicó también la emergencia de emociones como el alivio o la felicidad por terminar con una situación no deseada:

“*Encima, con la carga psicológica de decir ‘yo me quiero sacar esto’ y como que nadie te ayuda. No está bueno de parte del centro de salud, del sistema público de salud*” […] *Y cuando hice la interrupción sentí felicidad, es la verdad*” (Entrevista M, 30 años)

“*Necesitaba desesperadamente terminar con todo. Que se termine. Sentí alivio, felicidad cuando se terminó. Me saqué una mochila de encima tremenda*”. (Entrevista J, 35 años)

En los relatos se visibiliza fuertemente la apropiación del cuerpo como territorio a defender en el que se entronca con la autoafirmación subjetiva de *embarazo no deseado*. Esta autoafirmación subjetiva se vincula con el ejercicio de autonomía reproductiva en el que radica la capacidad de decidir por sí mismas: 

“*Para mí es la capacidad que yo tengo sobre mi cuerpo y que nadie me puede decir qué es lo que yo tengo que hacer*”. (Entrevista A, 26 años)

## Discusión y conclusiones

En este estudio nos propusimos tener un primer acercamiento a las implicancias subjetivas de mujeres adultas cisgénero que recurrieron a un aborto en Argentina con posterioridad a la sanción de la Ley 27610, que tuvieron algún tipo de vinculación con el movimiento de la “marea verde”. Entre los principales hallazgos se destaca que las mujeres participantes del estudio decidieron abortar, al considerar el embarazo como inesperado, sorpresivo e inoportuno para su momento vital, lo cual resulta relevante en términos del desafío a la maternidad como destino. Tal como plantean Fernández y Tajer^21^ y Rosenberg^15^ la decisión del aborto constituye un acto afirmativo que desestabiliza los mandatos de género patriarcales de la mujer=madre. Y es una oportunidad para las personas gestantes para elaborar diferentes conflictos en su propia biografía, pues la interrupción del embarazo puede detonar procesos de autonomía y agencia en los que ellas comiencen a priorizarse a sí mismas frente al histórico relegamiento^59^.

Si bien en los casos estudiados en este trabajo no se advirtieron narrativas conflictivas vinculadas a violencias de género en la pareja o en otros vínculos, como sostiene Rosenberg^14^^,^^15^ la decisión de interrumpir un embarazo puede implicar la posibilidad de poner límite a situaciones de sometimiento en las diferentes relaciones. Lo que también se aleja de las concepciones que ubican al aborto como un hecho ontológicamente traumático, tal como plantea la misma autora.

Asimismo, si consideramos que los procesos de subjetivación están imbuidos en lo histórico social y que la subjetividad comprende la apropiación siempre dinámica de ese escenario^51^, resulta consistente que estas mujeres, vinculadas a los feminismos en general o a la Campaña Nacional por el Derecho al Aborto Legal Seguro y Gratuito en particular, hayan podido decidir de manera autónoma sobre la interrupción y en compañía de sus amigas y, en algunos casos, parejas. Como plantea Tajer^43^, la legalización del aborto tuvo implicancias en la despenalización social de la práctica en Argentina, lo que redunda en salud mental colectiva y en la propia salud mental de las personas que abortan al promover una subjetivación más autónoma.

En relación con el acceso al aborto, los hallazgos del estudio coinciden con el trabajo de Romero *et al*.^60^ sobre el alcance en la información pública de los dos primeros años de implementación de la Ley 27610, en el que destacan los avances, las áreas que aún requieren atención y cómo se ha gestionado su aplicación en diferentes jurisdicciones^60^. También coincide con los trabajos de Lenta *et al*.^11^ y Tiseyra *et al*.^26^ quienes identifican obstáculos materiales (insumos, equipos y turnos) así como resistencias institucionales y profesionales vinculadas a prejuicios y posiciones antiderechos en el mismo período estudiado. Ello coincide con los problemas señalados por las mujeres de este estudio al momento de acceder al aborto en el sistema de salud. También, siguiendo la convergencia con los trabajos, se pudo observar la presencia de equipos de salud comprometidos con el derecho al aborto que apuntalaron las interrupciones, más allá de los problemas materiales. En general, se trata de equipos que trabajaban con consejerías integrales de salud sexual con posicionamientos feministas que problematizan la biomedicalización del derecho al aborto^34^.

También se hallaron cuestiones vinculadas a las persistencias y cambios en relación con los sentidos sobre el aborto y los afectos concomitantes. Resulta sugerente que, aunque el aborto sea legal en Argentina, persistían sentidos subjetivos vinculados al secreto y a cierta clandestinidad de la práctica, ya no desde el punto de vista jurídico como se observa en los trabajos de Rosenberg^39^, Sutton^61^, Discacciati^62^ y López y Carril^63^ que le otorgaban la calidad de bien prohibido^64^. En cambio, en este estudio, el sentido sobre lo silenciado o clandestino del aborto se desplaza hacia el orden de la experiencia de lo privado y lo no narrable por fuera del ámbito de máxima confianza para cada una de las participantes. Esto podría dar cuenta de que, a pesar de las transformaciones en el plano social general acerca de la despenalización, en el plano de lo íntimo insiste cierta sensación de que se trata de una cuestión que no resulta tan fácilmente de compartir ante el peligro de ser juzgadas por otras personas. De allí que persistan afectos como la vergüenza y la culpa por no haber evitado el embarazo no deseado o sorpresivo, que podrían relacionarse con el tabú sobre la sexualidad de las mujeres e, incluso, cierto castigo y estigmatización por su ejercicio de la sexualidad^65^. Esto nos lleva a resaltar que en el silencio sobre el tema se asientan las relaciones de poder de género patriarcal que aún siguen vigentes en la sociedad^66^. 

Al mismo tiempo, estos afectos advierten sobre la persistencia de las cargas de la anticoncepción y el cui­dado como responsabilidad de las mujeres^67^ ya que, aun cuando los varones aparecieron acompañando sus decisiones, no fueron in­terpelados en relación con sus papeles en el cuidado y la prevención del embarazo.

También emergió el miedo respecto de la práctica del aborto por la falta de información sobre el procedimiento de la interrupción en sí, el dolor y la muerte, en consistencia con trabajos de Rosenberg^14^^,^^15^^,^^39^, desarrollados en el período anterior a la legalización del aborto.

Alivio y felicidad fueron afectos relacionados con el logro de la interrupción. Como se mencionó más arriba en relación con los trabajos de Fernández y Tajer^21^ y Rosenberg^14^^,^^15^, estos hallazgos son consistentes con la posibilidad de que el aborto se constituya afirmativamente en una oportunidad para la toma de decisiones respecto de la propia biografía, lo cual puede resultar empoderante para estas mujeres. Pues ellas tomaron la decisión de abortar para seguir adelante con el proyecto de vida que venían desarrollando, en sintonía con los hallazgos de Espinosa Casanova^50^ con mujeres en otro contexto. Asimismo, en algunas de las entrevistas, la decisión también implicó una interpelación subjetiva en la que devienen procesos de reflexividad crítica sobre la propia vida y el cuidado personal. Es decir, transitaron por un proceso de subjetivación que habilitó cambios en las biografías que fueron más allá de la situación del embarazo y su interrupción^11^. Desde la epistemología de las emociones^54^, es posible señalar que esta reflexividad que se advierte en las narrativas resulta un recurso interpretativo que visibiliza claves acerca de la producción de significaciones y afectos a partir de la experiencia de aborto en un contexto de legalidad.

Para concluir, resaltamos que esta investigación se aproximó a los procesos subjetivos de siete mujeres que realizaron la práctica de la interrupción del embarazo en resonancia con el proceso histórico social vivido en Argentina y conocido como la “marea verde”. Sin embargo, algunas de las limitaciones del estudio podrían ser que la muestra es pequeña, que solo considera a mujeres cisgénero, excluyendo a otras personas con capacidad de gestar, y que las mujeres entrevistadas estaban vinculadas o apreciaban positivamente el activismo de la Campaña Nacional por el Derecho al Aborto. Pero la investigación cualitativa se centra en el abordaje profundo de casos que presentan interés intrínseco para descubrir significados, sentidos y sentimientos, por lo que la generalización no es un objetivo de la investigación^68^. No obstante, sí resulta pertinente profundizar en el estudio de las implicancias subjetivas del aborto con otros colectivos de personas gestantes, de diferentes edades, identidades de género, regiones y posicionamientos previos en relación con el aborto, a fin de ampliar las dimensiones de este trabajo.

Asimismo, el presente estudio aporta al campo de conocimiento de la subjetividad al adentrarse sobre el proceso que atraviesan las mujeres en la práctica de un aborto legal con su producción de sentidos subjetivos lo que ha permitido identificar significaciones y afectos relacionados con tal decisión en el escenario del aborto legal, así como su vinculación con la accesibilidad y el acompañamiento recibido. Estos hallazgos constituyen insumos claves para seguir trabajando en la plena implementación de la Ley 27610 de Acceso a la Interrupción Voluntaria del Embarazo desde las consejerías de salud sexual integral, como así también desde los activismos feministas que insisten en la desestigmatización y acceso pleno a esta práctica en Argentina y en la región.
